# A huge verrucous carcinoma of the lower lip reconstructed by double Abbe flap: A case report and literature review

**DOI:** 10.3389/fonc.2023.1086963

**Published:** 2023-01-27

**Authors:** Ke Chai, Jinbing Liu, Rong Xiao, Guiying Zhang, Yi Zhan

**Affiliations:** ^1^ Department of Dermatology, Second Xiangya Hospital of Central South University, Changsha, China; ^2^ Hunan Key Laboratory of Medical Epigenetics, Second Xiangya Hospital of Central South University, Changsha, China; ^3^ Department of Oral and Maxillofacial Surgery, Second Xiangya Hospital of Central South University, Changsha, China

**Keywords:** lip verrucous carcinoma, double Abbe flap reconstruction, differential diagnosis, decision optimization, case report

## Abstract

Lip verrucous carcinoma is a rare low-grade neoplasm, with an unsightly appearance and locally aggressive nature. Treatment of verrucous carcinoma is as yet challenging, no well-defined guidelines for effective and safe management of this disease are available. A case of a patient with a huge verrucous carcinoma of the lower lip successfully treated by surgical excision and double Abbe flap reconstruction technique is presented, and striking features of lip locations of this tumor as well as their management are discussed.

## Introduction

Verrucous carcinoma (VC) is a rare, well-differentiated variant of squamous cell carcinoma that can be separated into four subtypes according to the region of occurrence: oral type, plantar type, anogenital type, and other mucocutaneous sites (1, 2). Oral verrucous carcinoma (OVC) represents approximately 2–12% of all oral carcinomas (1, 3). As an uncommon disease, lip verrucous carcinoma typically presents as a verrucous, thick plaque on the lips resembling a cauliflower. The tumor grows slowly, it may cause tissue destruction but does not metastasize. The etiologies are not precisely known, which probably include smoking, betel nut chewing, and chronic inflammation (4, 5). The available results do not support a causal role of human papillomavirus (HPV) infection in the development of verrucous carcinoma (6–8). Treatment needs to be chosen with caution as the lesions are located on the face. We report a patient with a huge verrucous carcinoma of the lower lip, successfully treated by surgical excision and double Abbe flap reconstruction technique after failed non-surgical treatments.

## Case presentation

**Figure 1:**
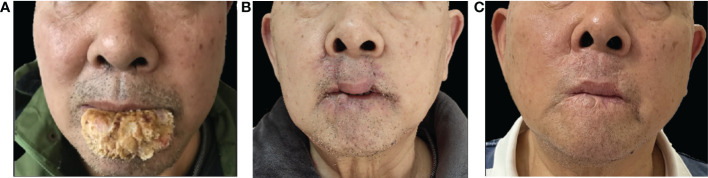
Clinical pictures: **(A)** Pre-operation image, an exophytic verrucous yellowish-to-whitish tumor occupying almost all the lower lip of approximately 5cm × 3cm^2^, resembling a cauliflower. **(B)** 30 days after surgical excision and double Abbe flap reconstruction, all skin lesions disappeared. **(C)** 30 months after surgery with a cosmetic appearance.

**Figure 2:**
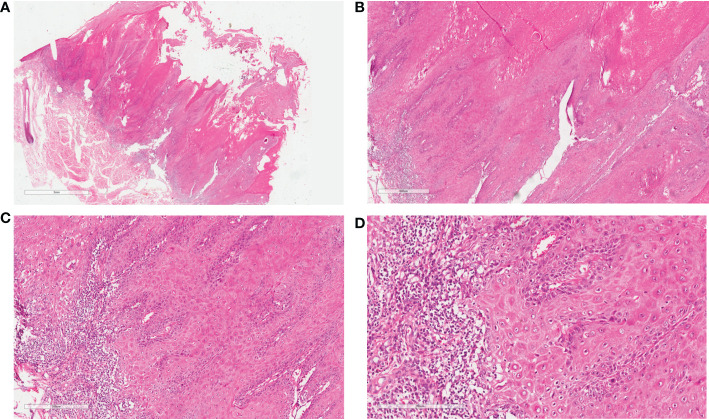
Pathological pictures: **(A, B)** Epithelial proliferation with verrucous appearance and significant keratosis, enlarged and fused endophytic epithelial ends with inflammatory infiltrate at the epithelial-stromal interface (H&E, ×10, ×40). **(C)** Endophytic papillae ending at different levels which forms pushing margins (H&E, ×100). **(D)** At high magnification, the basal cells are actively proliferating, and the spinous cells are enlarged in size with keratosis and red staining cytoplasm (H&E, ×100).

**Figure 3:**
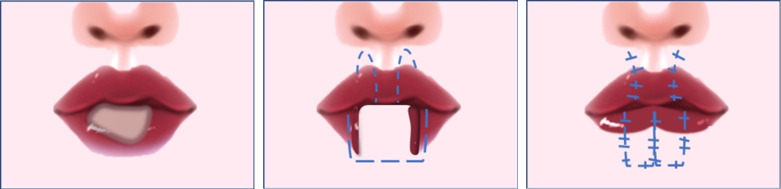
Reconstruction procedures: Rotating and insetting the double Abbe flap in the defect and suturing the distal edges of the two flaps to complete the repair of the defect.

A 67-year-old man presented with an enlarging verrucous tumor at his lower lip for 5 years. The patient smoked 10 cigarettes per day for 50 years and he was a heavy betel nut chewer. He noticed recurrent ulcers on his lower lip five years ago and applied topical application of spoiled egg white (a folk prescription) to the lesions on his own. After that, a hyperplastic warty mass gradually developed on the lower lip. The lesion was not accompanied by itching, pain or bleeding. Over the past 5 years, the lip lesion had been excised by cryosurgery or laser ablation on ten occasions. However, the disease recurred repeatedly. The patient recently visited our department with an exophytic verrucous yellowish-to-whitish tumor occupying almost all the lower lip of approximately 5cm × 3cm^2^ (**Figure 1A**). No analogous lesions were identified elsewhere in the body, and a clinical diagnosis of verrucous carcinoma was verified by the deep biopsy (**Figure 2**). Polymerase chain reaction (PCR) failed to support a relationship with HPV infection. Serology: hepatitis B was positive but inactive; syphilis, HIV, and hepatitis A were negative. Physical examination and further imaging did not give rise to the dissemination of the peripheral lymph nodes. Subsequently, the patient was referred to the department of maxillofacial surgery for further treatment. A wide local excision followed by the double Abbe flap reconstruction technique was performed by maxillofacial surgeons. Two symmetrical flaps with full-layer incisions were made on the external sides of the philtrum column apart for rotation and inset, with the vascular connection reserved at the distal end (**Figure 3**). The tumor was completely excised, and the histopathological result showed negative margins. After two weeks of evaluation, the double Abbe flap survived, and then the patient underwent the secondary procedure to separate the vascular pedicles. No relapse was observed more than 30 months after surgery, with a superior cosmetic result and nearly no limitation of mouth opening (**Figures 1B, C**).

## Discussion

Bad oral habits, including cigarette smoking and betel nut chewing, persistent lip ulcers, and the irritation of deteriorated foods may have contributed to the development of verrucous carcinoma of the lip in our case (1, 9). This disease is typically characterized by locally destructive behavior, with an unlikely tendency to metastasize, and appears as exophytic cauliflower-like, thick plaques on the lips (2). The most typical pathological features of VC are broad-based, well differentiated exophytic and endophytic epithelial proliferations with marked surface keratinization, without cellular atypia, invading the stroma with pushing borders (1). VC is comparable to many diseases in clinical and pathological conditions and deserves cautious differentiation, such as verrucous hyperplasia, squamous papilloma, and conventional squamous cell carcinoma (1, 10). Among them, squamous cell carcinoma, with varying degrees of cell atypia, has a potential proclivity to metastasize and carries a relatively dismal prognosis (10, 11). Although the prognosis of lip verrucous carcinoma is much better than other types of life-threatening malignancies, as a facially disfiguring disease, the particular location and the freakish appearance of lesions seriously affect patients’ mental health and quality of life. The choice of treatment modality is therefore crucial and clinicians strive to completely remove the tumor with normal tissue margins and to maximize preservation or restoration of lip function and appearance.

There are some treatment modalities reported to be applied, including surgery, cryosurgery, laser ablation, photodynamic therapy (PDT), radiation, chemotherapy, and immune response modifiers. Surgical excision is perceived as the primary treatment mode for verrucous carcinoma not exceeding 30% of the upper or lower lip. The radical treatment can simultaneously reduce the recurrence rate and avoid excessive lip defects (1, 12). Flap transplantation techniques are useful for repairing defects exceeding 30% of the lip length. However, inappropriate flaps may cause permanent deformity and limited function of the graft site (13, 14). Cryosurgery is commonly applied to minor or superficial lesions with a certain recurrence rate and associated side effects. Complaints about repetitive cryosurgery accompanied by pain or edema at the healing region are general (15). The laser ablation affords accurate resection, limited blood loss, as well as minimal contraction and scarring (16). But laser therapy also needs to be repeatedly operated, and a more extensive case series need to be explored (17, 18). Since the procedure of PDT is excruciating for regions with abundant nerve endings, there may be poor patient compliance in the application of lip disorders (19). Radiotherapy is debatable because of the potential for anaplastic transformation after irradiation (20, 21). Intra-arterial infusion chemotherapy facilitates the delivery of a high concentration of cancer medicine to the target region, inducing rapid tumor shrinkage and realizing local palliation within a relatively short duration (22). However, long-term chemotherapy may attract tumor resistance. In the meantime, it has been shown that immune response modifiers may be effective, such as imiquimod, which exhibits antiviral and antitumor effects *in vivo* (2). Even though multiple treatment modalities have been used for the treatment of verrucous carcinoma, surgical excision with adequate margins with or without reconstruction still remains the standard and widely adopted treatment.

Actually, no well-defined standards for safe and effective management of lip verrucous carcinoma are currently available. We present the related reports about lip verrucous carcinoma (**Table 1**) and summarize the advantages and disadvantages of various treatment modalities (**Table 2**). Our patient had undergone ten previous unsatisfactory non-surgical regimens, including cryosurgery and laser ablation, which could be partly attributed to incomplete lesion eradication. After a comprehensive evaluation by the dermatologist and maxillofacial surgeons, considering that the recurrence predicts a poor prognosis, the decision was made to conduct a surgical intervention. Restoring wide defects caused by lip carcinoma resection (commissure was not involved) was a challenge for surgeons. However, the use of conventional flaps, which are used for defects of two-thirds or even more of the lip length may result in the formation of deformity. For example, a smile deformity or a “fish-mouth” may accompany with the use of Bernard and Paramaniac flaps, especially in edentulous patients (39). Maxillofacial surgeons designed two Abbe flaps symmetrically on the upper lip to restitute the lower lip defect, named as the double Abbe flap. The double Abbe flap can better maintain the lip shape with the bilateral symmetrical design and a small auxiliary incision. Combined with two flaps, the novel reconstruction technique is suitable for large-area lip defects, which avoids some major trauma or postoperative complications caused by other single larger adjuvant incisions (14, 39). Overall, this reconstruction technique has less influence on the lip shape and provides adequate mouth opening without dysfunction in our case.

**Table 1:** Summary of the 22 patients with verrucous carcinoma of the lip.

No	Age	Sex	Location	Size	Metastasis	Possible etiologic factor	HPV infection	Therapy	Follow-up
1	34	F	Lower lip	1.5 × 1.8 × 0.9cm^3^	No	High-level hormones during pregnancy	No	CO_2_ laser ablation	No recurrence for more than 21 months (16)
2	56	M	Lip angle, buccal mucosa	3 × 2.5 × 1 cm^3^; 6 × 4.5 cm^2^	No	Cigarette smoking and betel nut chewing	Uncertain	5-aminolevulinic acid-mediated photodynamic therapy	No recurrence for more than 6 months (23)
3	80	M	Lower lip	2.5 × 1.3 cm^2^	No	Uncertain	Uncertain	Local bleomycin by iontophoresis	No recurrence for more than 6 months (24)
4	75	M	Lower lip	No record available	No	Cigarette smoking	Uncertain	Cryotherapy, local application of imiquimod cream	No recurrence for more than 30 months (2)
5	70	M	Lower lip	4 cm in diameter	No	Cigarette smoking and alcohol	Uncertain	CO_2_ laser ablation, local application of imiquimod cream	No recurrence for more than 30 months (2)
6	54	M	Lip, buccal mucosa	Entire lower lip, part of the left upper lip	No	Cigarette smoking and betel nut chewing	Uncertain	CO_2_ laser ablation, systemic retinoic acid	A suspicious regrowth of the tumor was detected on week 7 (17)
7	68	F	Lower lip	10 × 5.5 cm^2^	No	Cigarette smoking and betel nut chewing	Uncertain	Intra-arterial infusion of methotrexate	The nonhealing ulcer was noted again after 16 months (22)
8	57	M	Lower lip	No record available	No	Cigarette smoking	Uncertain	Mohs micrographic surgery	No record available (25)
9	45	F	Lower lip, mouth angle	No record available	No	Cigarette smoking, HIV/HPV coinfection	Yes	Surgical excision	The lesion recurred 3 times in the next 4 years (26)
10	70	F	Lip mucosa	No record available	No	Cigarette smoking	Uncertain	Surgical excision	No recurrence for more than 2 years (4)
11	59	M	left upper lip	2.8 × 0.7 × 0.4 cm^3^	No	Cigarette smoking, cutaneous horns	Uncertain	Surgical excision, CO_2_ laser ablation	No record available (27)
12	71	F	Lower lip, mandibular alveolar, buccal mucosa	1.5 × 1.3 cm^2^	No	Oral lichen planus, HCV infection, hyperinsulinemia	Uncertain	Surgical excision, radiotherapy	No recurrence for more than 2 years (28)
13	75	F	Left lower lip	1.4 × 2.0 cm^2^	No	Uncertain	Uncertain	Surgical excision, V-Y advancement flap and cross-lip vermilion flap	No recurrence for more than 1 year (29)
14	91	F	Middle lower lip	1.5 × 1.0 cm^2^	No	Uncertain	Uncertain	Surgical excision and tongue flap reconstruction technique	No recurrence for more than 1 year (30)
15	84	M	Lower lip, commissure, buccal mucosa	4 × 3 cm^2^	No	Cigar smoking, tobacco chewing, and unmatched dentures	Uncertain	Surgical excision, CO_2_ laser ablation and split-thickness skin grafts reconstruction technique	No recurrence for more than 4 years (31)
16	80	F	Upper lip, buccal mucosa	4 cm in diameter	No	Uncertain	No	Surgical excision, Karapandzic-modified flap reconstruction technique; CO_2_ laser ablation	No recurrence for more than 2 years (18)
17	67	F	Lower lip	5 × 3cm^2^	No	Cigarette smoking and betel nut chewing	No	Surgical excision and double Abbe flap reconstruction technique	No recurrence for more than 30 months (the present case)
18	55	M	Right commissure of the lip	No record available	No	Measles infection and noma	Uncertain	Surgical excision and forearm flap reconstruction technique	No record available (32)
19	70	F	Left lip, mouth angle, buccal mucosa, outer mental skin	No record available	No	Uncertain	Uncertain	Surgical excision, SAIF flap reconstruction	No record available (33)
20	50	F	Lip, commissure, buccal mucosa	Whole lower lip, part of the upper lip	Uncertain	Tobacco chewing	No	Surgical excision with bilateral neck node dissection, ALT flap reconstruction technique	No record available (34)
21	55	M	Lip, buccal mucosa	16 × 8 cm^2^	Yes	Cigarette smoking	No	Surgical excision with bilateral neck node dissection, ALT flap reconstruction, scrotal skin grafting, autologous fat grafting, skin tattooing	No recurrence for more than 2 years (35)
22	72	F	Lip and palate	Diameter from 1.5 cm to 3.5 cm	Yes	Actinic cheilitis	Yes	Untreated	Died of deterioration before treatment (36)

**Table 2:** Summary of benefits and limitations of treatments used in Lip Verrucous Carcinoma.

Treatments	Benefits	Limitations
Cryosurgery (15)	Suitable for minor or surface damage.	Repetitive operations due to the high recurrence rate. Severe pain or obvious edema.
Laser ablation (16–18)	Accurate resection, limited blood loss, and minimal contraction and scarring. As a tumor debulking therapy before surgery.	Complete resection is difficult to ensure. Relatively high recurrence rate.
Photodynamic therapy (19)	A minimally invasive and negligibly toxic technique. High efficacy. Little or no scar formation.	The procedure is excruciating for regions with abundant nerve endings.
Radiation (20, 21)	Suitable for the advanced tumor stages which are not an indication for surgery.	Radiation involves surrounding normal tissue. Radiation promotes anaplastic transformation.
Intra-arterial infusion chemotherapy (22)	Facilitating the delivery of a high concentration of cancer medicine to the target region. Inducing rapid tumor shrinkage. Realizing local palliation within a relatively short duration. As a tumor debulking therapy before surgery.	Expensive modalities. Lack of adequate information.
Immune response modifiers (2)	Exerting antiviral and antitumor effects.	Corrosion of the skin. Difficulty adhering to treatment.
Simple surgical excision (12–14)	Rare tumor recurrence and good prognosis. Suitable for verrucous carcinoma not exceeding 30% of the upper or lower lip.	Insufficient to repair large defects.
Surgical excision with flap reconstruction (14, 37, 38)	Reconstructing large defects after lesion resection: < 30% of the lip: V-Y advancement flap. 30%-65% of the lip: Abbe flap, Abbe-Estlander flap, Bernard flap, Karapandzic flap, etc. 65%-80% of the lip: double Abbe flap, Johanson staircase flap, Karapandzic flap, Bernard-Webster technique, etc. > 80%-85% of the lip: double Abbe flap, Bernard-Webster technique, Gilles fan flap, etc. A massive resection of the lip, chin, and mandible: ALT flap, radial forearm flap, etc.	Long operation time and large wound surface. Unsightly cosmetic consequences. Scar hyperplasia may limit mouth opening.

It seems that the curative effect of this therapy is excellent at present, as the patient remains asymptomatic 30 months after surgery. There is currently no report regarding the use of double Abbe flap reconstruction techniques for lip verrucous carcinoma defects as we know. The authors report a case of a rare tumor with a relatively large size and bizarre appearance. The interest of this case is to gather previous reports to present the striking features of lip verrucous carcinoma: epidemiology, cosmetic features, peripheral dissemination, pathophysiological determinants such as human papillomavirus infection, treatment modalities, and follow-up results. Furthermore, the reporters summarized various treatment modalities and their advantages and disadvantages to supply additional clues during clinical assessment and optimize the clinical benefits for patients. In addition, it is worth emphasizing that the double Abbe flap may be a useful and promising approach in the reconstruction of the lower lip following large-area verrucous carcinoma resection, as it helps to completely remove the tumor while maximizing the preservation or restoration of lip function and cosmesis.

## Data availability statement

The original contributions presented in the study are included in the article/Supplementary Material. Further inquiries can be directed to the corresponding author.

## Ethics statement

Written informed consent was obtained from the individual(s) for the publication of any potentially identifiable images or data included in this article.

## Author contributions

KC drafted the manuscript, drew the figures, and summarized the table. JL, RX, and GZ discussed and revised the manuscript. YZ designed the study, reviewed and edited the paper. All authors contributed to the article and approved the submitted version.
